# Supplementary material for: Factors associated with poor adherence to intrapartum fetal heart monitoring in relationship to intrapartum related death: A prospective cohort study
Source: PLOS Glob Public Health. 2022 May 23;2(5):e0000289. doi: 10.1371/journal.pgph.0000289 (PMC10021382; doi:10.1371/journal.pgph.0000289)
Supplement: Attachment — Submitted filename: plos gh_response letter.docx [file pgph.0000289.s003.docx]

2022-01-11

**Response to editorial comment**

**Comment 1#.** Please use the space provided to explain your answers to the questions above. You may also include additional comments for the author, including concerns about dual publication, research ethics, or publication ethics. (Please upload your review as an attachment if it exceeds 20,000 characters)

**Response-** Thank you for inviting us to resubmit a revised manuscript based on the reviewer’s comment. This is not a dual publication. Ethical approval has been taken from ethics committee for the research.

**Response for first reviewer’s comment**

**Comment 2#.** Second, the study needs some minor revision from English and grammar perspective, for example" This study used data from a period of 18 months- instead you can write the data had been collected over 18 months time frame between April 2017 and October 2018 "

Response. We have now improved the grammatical errors.

**Comment 3#.** Third, you included bar graph and P value chart, I would suggest including them in the body of the study to make the reader understands better the data instead of writing the at the end on the study.

Response- We have added the bar graph and p-value in the main text.

**Comment 4#.** Forth, the declaration is written at the end of the study, I would advise to write it before the introduction of the study

Response- We have now moved the declaration section before the introduction of the study.

**Response to second reviewer’s comment**

**Comment 5#.** The title is good and it reflects the authors context of the problem phenomenon. The background information is substantial enough. The methodology is adequate, however, there was a comparison stated in the result aspect which the methodology didn't state if there is a comparison group to the cohort group.

Response- We have revised the title of the publication. We have now added the comparison made between the group in the cohort is added in the methodology section as “we made the comparison on the coverage of standard FHRM and sub-standard FHRM by …..”

**Comment 6#.** The result is good. Data was rightly processed analysed. However, the logistic regression to establish the association was not clearly seen. There is a disconnect also between the title and the conclusion aspects. The major objective of the study was establish the association between fatal heart rate monitoring and intrapartum related death so focus should be on analysing data and use as evidence to accept or reject hypothesis that guides the study.

Response. We have now aligned the title and the conclusion as well as presented the logistic regression in a better way.

**Response to third reviewer’s comment**

Major essential revisions:

**Comment 7#.** In method, the hospital selection criteria were not found, as well, the inclusion and exclusion criteria for selecting the participants were not backed up with supporting literatures by in texted citations which may create barriers in understanding the validation and lessens the chances of recreating it in a similar setting.

Response- We have now added the hospital selection criteria in study design sub-section as “The 12 public hospitals were selected to represent the public referral hospital in each province of the country”. The inclusion and exclusion criteria is provided in the participant sub-section as “Women with absence of FHS upon admission and antepartum stillbirth were excluded. All women who consented to participate in the study were enrolled and were followed up upon discharge from the hospitals.”

**Comment 8#.** How the randomization was assured at the time of sample selection was not mentioned. The data collection and management section was not clearly and elaborately explained. On what basis the data for FHRM variables and birth outcome were extracted from the medical records? As it’s a follow-up study, was the cohort being followed up on a daily basis or the data for the total period was collected at once? If the second one was applied, would it be considered as a prospective cohort or a retrospective cohort? Bit more detailed explanation of the data collection might come in handy to grasp the method.

Response- The random selection of the hospital in each wedge is mentioned in the study design section. We have further elaborated the data collected procedure and the nature of data collection in the data collection and management section as “The clinical research nurses were stationed in the labour and delivery and postnatal unit all around the clock throughout the study period. After the woman was admitted in the labour room were assessed by eligibility by the clinical research nurse. Woman who was eligible to the study was approached by research nurses to be enrolled in the study. Woman enrolled in the study were followed from the time of admission until discharge from the hospital. The research team collected woman’s medical information from medical records and socio-economic information through semi-structured interviews at discharge in a paper-based form. The research team extracted the clinical information on fetal heart rate monitoring, obstetric progress, birth outcome of each enrolled participants at the labour and postnatal unit. The social and demographic information was collected from semi-structured interview. The research coordinator assessed the quality of data collection and ensured the completeness of data collected."

**Comment 9#.** About the data management, was the data only cleaned using Survey Processing System (CS-Pro)? Moreover, was all the data managed at once using this software package and that also at the end of the follow up period or maintaining a basis (daily/weekly/monthly)? These information should be elaborative in a cohort study.

Response-We have further elaborated the data cleaning and management process as “During the weekdays, the research coordinator from each study site, compiled all the filled-up forms and sealed them in a closed envelop and sent it through designated courier service at the central research office in Kathmandu. The received forms from each side were assessed for completeness by a data management team at the central research office in Kathmandu. Followed the assessment, the paper-based form was into the database by the data entry and management team every data. All the entered paper forms were indexed according to hospital. The database manager cleaned all the entered forms using the Survey Processing System (CS-Pro) on a monthly basis.”

**Comment 10#.** In measurements, the source of outcome measure definition should be mentioned. The absence of such, results in invalidation, hence stating the outcome variable of the current study undefined. The main exposure variable definition was not supported by any citation where the WHO standard for the mentioned statement “Adherence to FHRM protocol during labor was deemed to be adequate when the FHR was monitored as per protocol at least every 30 minutes during the first stage of labor and at least every 15 minutes during the second stage of labor according to WHO standards” could be found. Not a single mention of the non-exposed group, neither the definition of non-adherence to FHRM was found. Hence, the measurement for depicting sub-optimal FHRM is unstated and undefined. None of the variables’ measures except for the “Obstetric complication upon admission” were validated with either reliable or any resources. Such actions could be addressed as invalidation of a manuscript.

Response- We have now provided the reference to the source of outcome.

**Comment 11#.** The mention of significance level value which would be accepted could be mentioned. The ethical consideration section should be present in the method too.

Response- We added the ethical consideration in the method section.

**Comment 12#.** In the flowchart, there was no mention of sample dropout, a mention of “no sample dropped out or died while following the cohort” might dispel any scope of measurement bias.

Response- We did not imply to say that all mortality events occurring in the hospital were captured, rather all the mortality event collected in the study was cleaned and analysed. We have removed the sentence now.

**Comment 13#.** In result, mention of tests for descriptive analysis could be more appropriate and inferential statistical models e.g., bi-variate logistic regression may not be appropriate for descriptive statistics. By what type of tests “The bi-variate association between intrapartum related death with primary exposure-FHRM and other exposures” - was assessed? It should be stated clearly.

Response- We have now provided the test used for descriptive analysis in “analysis” section.

**Comment 14#.** The objective did not mention of more than one outcome, therefore the mention of “multi-variate” association is not accepted as there’s a difference between multivariable and multivariate outcome.

Response- We have removed the word “multi-variate” and mentioned as association between outcome and exposure.

**Comment 15#.** Coming to the 3 models mentioned here, it says, “Model I was done by adjusting the primary exposure with antepartum related variable”. It would be helpful to assess the process if could know what the antepartum related variables exactly in this study are, as those were not found mentioned in the individual sub section under the method section, moreover, the antepartum deaths were one of the exclusion criteria.

Response- We have now added sentences on the types of variables that were adjusted in model I, II and III as “For model I, demographic and antenatal variables were adjusted. Demographic and antepartum variables were maternal education, maternal ethnicity, maternal age, parity, antenatal check-up from skilled provider. For model II, intrapartum variables were adjusted. Intrapartum variables were obstetrics complication during labour, multiple pregnancy, mode of birth, preterm birth, low birth weight and sex of baby. For model III, demographic, antenatal and intrapartum variables were adjusted.”

**Comment 16#** “A total of 209 intrapartum stillbirths and 668 early neonatal deaths during the study period, with intrapartum related mortality rate of 10.5 per thousand deliveries. (Figure 1) The probability of intrapartum related mortality varied by hospital on a scale from 5 – 26 per 1000 birth. (Figure 2)” - Stating sentences like these within a para, is really unstructured and not actually depicting anything, it is near to suitable for a legend of a figure. Again, no mention of such values (209, 668) was observed in figure 1.

Response- We have now revised the introductory sentence in result section.

**Comment 17#.** “More than 60% of the women delivering in Lumbini, Bheri, Koshi and Lumbini hospital had sub-optimal FHRM.” The data illustrated from figure 3 showed more than 75% of women delivering in Lumbini, Bheri having sub-optimal FHRM as opposed to Koshi having less than that indicating the need for better elaboration of data presentation.

Response. We have now revised it as “More than 75% of women delivering in Lumbini and Bheri hospital had sub-optimal FHRM”.

**Comment 18#.** Why assessing the odds by the variables’ crude values instead of observing by their adjusted values? This leaves the scope of confounding bias as the tentative confounders are not adjusted here in table 2, hence the results do not hold a great significance probably.

Response-It is important to provide the unadjusted association of the primary exposure and other exposure variables with primary outcome.

**Comment 19#.** Besides, no notes under the tables mentioning abbreviation of terms, (e.g., aOR, cOR) and how the p-value was set as significant were found. Again, the contents of the table 2 was not labeled with their respective headings properly (e.g., what does “Yes, 37011 (44.2%), No, 46698 (55.8%) as the heading of table 1 or Alive (82,832, 99.0%), Intrapartum related death (877, 1.0%) as the heading of table 2” such headings mean?). Also, what are the percentage presented in the above-mentioned examples indicating? Are they prevalence? Or, are they presenting only the percent value of the distribution here? A bit more emphasis could be given in the result and the method sections as these are two of the most important sections of a scientific report.

Response- We have provide the full form of cOR and aOR in each table. The percentage presented in table 2 is the incidence of outcome for each exposure variables.

**Minor essential revisions:**

**Comment 20#.** The introduction writing format lacked global perspective and strengthened justification. Introduction could be more sufficed. The study objective might be justified enough if it were cited with more evidences from related studies across the LMICs around of South Asian countries. Again, lack of global evidences from studies weakened both the purpose and background of the study. Also, elaborated information related to the main outcome could have been included.

Response- We have now thoroughly revised the first four paragraph of the introduction section.

**Comment 21#.** In method, the necessity of five data sets and the function of MICE method should have been explained. The data created after the sensitivity analysis gathered in a supplementary table could have been included along with the submission, exemption in which does not really hold the statement any longer meaningful. STROBE, TRIPOD or CONSORT checklists should have been followed for the data reporting as its highly recommended.

Response. The MICE function provides the systemic way to impute the missing values. We have now provided the filled in STROBE checklist.

**Comment 22#**. In result, “Descriptive analysis of intrapartum related death by hospital and coverage of FHRM by hospitals”, a structured sentence opposed to this is much preferred.

Response- We have now revised the sentence structure

Discretionary revisions:

**Comment 23#.** Few grammatical corrections, uses of preposition and word selection may result in greater outcome for the readers’ understanding. In the end, unfortunately, to me, the conclusion is not adequately supported by the definition provided in the methods and strengthened by the data provided in some of the tables and figures. These sections could be revised.

Response- We have now improved the conclusion.

Recommendations:

**Comment 24#.** The study concept in itself is alarming, the large number of sample size is also a significant point, but the conceptual framework could be more suitable, brief and accurate by following the reporting guidelines of scientific report writing and the findings could be summarized along with the implementation ideas backing up the intervention applied.

Response- We have now added the implementation idea to improve the adherence to fetal heart rate monitoring in the discussion section in lines XX-XX as “Improving adherence to fetal heart rate monitoring during labour in these hospital despite a national standard for care will require quality improvement implementation approach. A contextual analysis in each hospital on the bottleneck for adherence to care followed by continuous planning, implementing and review of progress. Adequate adherence to fetal heart rate monitoring will require adequate monitoring device, trained and adequate human resource, continuously measuring the coverage of care and periodic review meeting on the progress of implementation. Furthermore, the hospital system needs improvement in timely decision for instrumental or cesarean section for high-risk fetus.”

**4^th^ reviewer’s comment**

**Comment 25#.** I would urge the authors to revise the definition of parity used in this paper (parity vs gravity) as at present their terminology is confusing. All definitions could be put in a table.

Response- We did not change term “parity” into “gravity” as we include only previous viable pregnancy.

**Comment 26#.** It is plausible that the degree of prematurity (<28, 28-32,>32 weeks) could influence the outcome. Could the authors explain why it was used as binary rather than categorical.

Response- We agree that the odds of mortality higher with severity of prematurity. However, we know that prematurity, less than 37 weeks has risk for mortality, so we did not categorize into different prematurity group.

**Comment 27#.** Could you please remove the p values from table 1, as hypothesis testing does not seem appropriate. Table 2, why was age group 26-30 used as baseline? The age group 19-25 makes more theoretical (less risk) and statistical (larger category/ more stable model) sense. Again, revise the use of parity terminology and consider preterm as categorial. Sex: M/F more appropriate.

Response- This is not an intervention study, where in the population characteristic in baseline and intervention period is compared without p-value. Table 1 compares the coverage of fetal heart rate monitoring among the different co-variate. In table 2, we used age group 26-30 yr group as reference based on the low incidence of mortality in this group. We have now revised boy/girl as male/female.

**Comment 28#.** Could the authors conduct sub-group analysis for women who had complications during admission to help further stratify the risk (maternal vs fatal complications).

Response-The obstetric complication during admission is maternal related with very few number of fetal complication.

**Comment 29#.** Definition of who companions were would help contextualise the findings (family? doula?)

Response- The companion during birth are the family members not doula.

**Comment 30#.** Hart’s law of inverse care is very useful concept here, the discussion would benefit from few more sentences to explain this point better.

Response- We have now revised the sentence on Hart’s law of inverse care related to proportion of sub-optimal fetal heart rate monitoring.

**Response to fifth Reviewer’s comments**

**Comment 31#.** The title does not reflect the contents of the paper accurately. The title can be best written as “factors associated with poor adherence to intrapartum FHRM in relationship to intrapartum related deaths”

Response- We have revised the title as “Factors associated with poor adherence to intrapartum fetal heart monitoring in relationship to intrapartum related death: a prospective cohort study”

**Comment 32#.** To the best of my understanding: this is not a cohort study rather a “prospective observational study”. The time of follow up is only during labor and that cannot be termed at Cohort

Response- This is a prospective cohort study and not a prospective observation study. We did not observe all birth, the research nurses enrolled the women prospectively since the time of admission in labour room and followed the cohort until discharge.

**Comment 33#.** There is term “admission” is consistently used but it is not specific where are the pregnant women admitted is it labor ward? Antenatal ward? Theatre or where? Authors need to be specific as admission to labor ward is different from admission to ANC ward for example

Response- The term “admission” is admission in labour room. We have clarified in throughout the document.

**Comment 34#.** The term coverage is used vaguely throughout the document. It is better to be specific, use “proportional of FHRM monitored per protocol” or percentage of adherence. Coverage should not be used

Response- Agree, we have replaced the word coverage with “proportion of FHRM monitored per protocol”.

**Comment 35#.** The paper has two main aims and need to be clarified:
• Predictors of suboptimal FHRM
• Association of sub-optimal FHRM and intrapartum deaths
These two are not being specific in your paper

Response-We have now clearly specified, the two main objective as “the study aims to assess the predictors of suboptimal fetal heart rate monitoring and assess the association of sub-optimal FHRM and intrapartum related deaths”

**Comment 36#.** I am not a statistician, but I was trained to use the term “likelihood” with caution. In the abstract conclusion the term likelihood stands for “odds” and these two must not be used interchangeably. Likelihood is used when you are comparing the two models and you have a ratio with p-value comparing the models.

Response- The odds ratio is presented as odds or likelihood of mortality associated with particular variable.

**Comment 37#.** Introduction: The first sentence is not clear please re-write. Overall, the introduction is too much focus on the literature from the same study group (papers referred) and Nepal. Authors did not take time to search for available evidence from other scientists across the world especially in Africa and specifically Tanzania, Uganda, and DR Congo where similar interventions are being implemented. I advise to re-write this section

Response- We have now thoroughly revised the introduction section and have referenced the other studies done in Africa and Asia.

**Comment 38#.** Methods. Has NePeriQIP study been published (I think the answer is Yes). If ye,s don’t the authors see that these data have been published and if Not, then the evaluation of the project will be duplication of efforts? Was this study described in the protocol or it just emerged?

Response-Yes, the primary outcome of NePeriQIP has been published. In this study, we did not evaluate effect of NePeriQIP on fetal heart rate monitoring, so this is not a duplication of effort rather understanding the association of sub-optimal fetal heart rate monitoring on intrapartum related mortality.

**Comment 39#.** What was the instrument used to measure FRH? This is important because different devices has different levels of accuracy

Response- Auscultation or pinard was the instrumental used to fetal heart rate.

**Comment 40#.** Why was GA be estimated by LNMP instead of adding more robust methods such as use of 1st trimester Ultrasound? Pre-term labor; what if the GA was unknown, how was that established?

Response- Gestational age is estimated based on last menstrual period.

**Comment 41#.** Statistical analysis. Why was multi-level modelling done? It is not clear in the manuscript how was this done and for what purpose The use of this methods would benefit by adding a simple formula (stata syntax/code) within the text

Response- The multi-level modelling was done to separately adjust the antenatal and intrapartum factors and finally adjust all the factors together with intrapartum related mortality. The antenatal factor might have different level of association with mortality than the intrapartum related factor, so adjusting them together into one bundle might mask the risk of sub-optimal FHRM association with intrapartum related mortality.

**Comment 42#.** Why was MAR used as the imputation methods and no other methods?

MAR was used to provide the values which might have been missed to be gathered randomly especially for birth weight and gestational age.

MAR is used if there is any systematic difference between the missing values and the observed values. In this study, there was a difference in mortality among those who had missing gestational age and birth weight than those with measure, so we did the MAR imputation method to generate missing values.

**Comment 43#** Results: In the first paragraph, Why were the hospitals classified

Response- We have not categorized the hospitals, rather presented the hospital with highest proportion of sub-optimal FHRM.

**Comment 44#.** Reference: Most of the reference are from same study group. Authors are advised to seek evidence elsewhere to make their study robust

Response- We have added new reference from Asia and Africa.

**Comment 45#.** Figure 1: Authors did imputation to counter missing values but there still missing data, why? Why was this missing information not imputed?

Response- We could not impute some missing values for birth weight and gestational as we could not get the reference to impute. For example in some cases both birth weight and gestational age was missing, so those could not be imputed to generate the value.

**Comment 46#.** Figure 2 is missing

Response- Figure 2 is mistakenly labelled as figure 3.

**Comment 47#.** Figure 3: probability of intrapartum related deaths. Change label from probability estimate to “point estimates”

Response- We agree that it is a point estimate not probability. We have changed the label

**Comment 48#.** There is a lot of redundant information. Authors should decide either to use point estimates and CI in table or just the lines with point estimates and Cis embedded within but not both as same information is presented twice and this will confuse the reader

Response- We have removed the redundancy to the extent possible.
